# Human-Wildlife Conflict in Save Valley Conservancy: Residents' Attitude Toward Wildlife Conservation

**DOI:** 10.1155/2022/2107711

**Published:** 2022-04-28

**Authors:** Peter Makumbe, Stenly Mapurazi, Sostina Jaravani, Isaac Matsilele

**Affiliations:** ^1^Shangani Ranch, P. O. Box 24, Shangani, Zimbabwe; ^2^Department of Natural Resources, Bindura University of Science Education, Private Bag 1020, Bindura, Zimbabwe; ^3^Chiredzi Rural District Council, Chiredzi, Zimbabwe

## Abstract

Human settlement in protected areas (PAs) is a major conservation concern in developing nations as it fuels human-wildlife conflicts (HWCs). The objectives of this study were to (i) determine the key wildlife species causing conflict, (ii) assess the perceptions of residents toward the major causes of conflict with wildlife, and (iii) evaluate the attitudes of residents toward problem animals. We conducted face-to-face semistructured interviews and two reconnaissance field surveys with 290 respondents residing in Save Valley Conservancy (SVC), in Southeast Lowveld Zimbabwe from January 2014 to June 2014. Results showed that lions (*Panthera leo*), spotted hyenas (*Crocuta crocuta*), elephants (*Loxodonta africana*), and Nile crocodiles (*Crocodylus niloticus*) were the major animals involved in the conflict. Our results also showed that the land-use change from wildlife ranching to farming and contested land ownership were perceived as the major causes of HWCs. Respondents who had lived in the area longer were more likely to agree that change in land use (Ordinal logistic regression: *B* = 1.32, Odds Ratio (OR) = 3.74) and contested land ownership (*B* = .67, OR = 1.95) were major sources of conflict. In addition, increased encounters between people and wildlife triggered mixed attitudes toward problem animals. For example, males were less likely to have a negative attitude toward problem animals compared to females (Multinomial logistic regression: *B* = −1.39; OR = .25). Residents who had stayed for less than five years were more likely to have a negative attitude toward problem animals than those who had stayed longer (*B* = 3.6; OR = 36.71). These results suggest that there is a need to relook at the resettlement pattern because coordinating HWCs and implementing sustainable conservation objectives are easy in a well-planned settlement. Stakeholders need to come together and create awareness of the use of HWCs mitigations measures.

## 1. Introduction

The relationship between wild animals and humans is complex, ranging from positive aspects such as appreciation, reverence, and acceptance to negative aspects such as retaliation and killing. The antagonistic relationship between humans and wildlife has become known as the human-wildlife conflict (HWC). Moreover, humans have always hunted animals, while wildlife species are crop raiders or livestock depredators [1]. HWC is of great concern worldwide because it has both positive and negative impacts on biodiversity and human populations [2–4]. In addition, one or more human-wildlife encounters can strongly influence people's attitudes, shaping their responses to future interactions with specific wildlife species.

HWCs can take a variety of forms, including attacks on humans, depredation [4, 5], and crop-raiding [6, 7]. Taxa that can cause many problems include medium to large body-sized herbivores and carnivores, as well as primates [7, 8]. Communities near protected areas (PAs) suffer from crop-raiding and livestock predation, which is often the biggest cause of conflict in Africa [7]. People residing inside PAs are even more exposed and vulnerable to problem-causing wild animal species [9, 10]. In Ethiopia, Tamrat et al. [11] concluded that livestock predation is intense in and around a protected sanctuary. Similarly, Matseketsa et al. [5] demonstrated that elephants (*Loxodonta africana*), lions (*Panthera leo*), and spotted hyenas (*Crocuta crocuta*) are a problem in communities bordering Save Valley Conservancy (SVC) in Zimbabwe. Thus, people living in and around PAs often suffer the most from problem animals.

Numerous studies on HWCs have been conducted in Africa (e.g., [5, 7, 8]). The consensus is that the successful conservation of wildlife species is closely linked to people's perceptions and attitudes toward wildlife. There is a growing recognition among conservationists that effective wildlife management must be based on public attitudes toward wildlife [12, 13]. Ignoring social, cultural, and political dimensions while focusing only on the ecological impacts of wildlife presents a major challenge in addressing HWC-related issues [14]. In addition, the costs associated with wildlife can lead people to have negative attitudes and perceptions toward wildlife conservation [15]. For example, wildlife attacks on humans and crop-raiding can trigger negative perceptions, and people may respond with retaliatory killings or support of killings by others [16, 17]. Moreto [17] showed that, in Uganda, community members who suffered a sudden loss became frustrated and angry and responded by killing the animal that caused the harm. Similarly, Viollaz et al. [18] showed that people who lose their livestock to leopards (*Panthera pardus*) responded by killing the animal in 9 out of 10 cases. Consequently, understanding residents' attitudes toward problem animals is a critical guide to successful wildlife species conservation [12]. Any successful solution for HWCs must therefore directly address human perceptions of wildlife.

Many PAs in Africa and worldwide have been practicing “fortress conservation.” Fortress conservation or the protectionist approach is characterized by three principles: (i) local people who are dependent on the natural resources are excluded from PAs, (ii) enforcement (i.e., exclusion of people from PAs) is achieved by deploying park rangers who patrol boundaries using “fines and fences” approach to ensure compliance, and (iii) only tourism, safari hunting, and research are considered appropriate uses within the PAs [19]. In this model, wild animals can freely roam outside PAs in the process of inflicting damage on communities. On the other hand, people are denied access to resources inside the PAs. Because people living near PAs are seen as criminals, poachers, or squatters on the land that they occupied for a long time, they tend to be antagonistic toward fortress-style conservation initiatives and are more likely to have negative attitudes toward wildlife species. Studies have shown that the protectionist approach has failed to some extent in wildlife conservation in Africa and globally [20, 21]. For example, Duffy et al. [22] argue that the militarization of conservation has had negative impacts, such as an increase in poaching and the remembrance of past injustices. The complete exclusion of people from conservation issues through security fences and the imposition of fines for wildlife-related offenses in the 1970s and 1980s [23] led to a growing recognition that conservation works best when local communities affected by wildlife are involved in the management process.

This perceived injustice of natural resource privatization partly motivated the introduction of the Fast Track Land Reform Program (FTLRP) in 2000. The FTLRP was an extreme land redistribution measure, as the government of Zimbabwe forcibly evicted white commercial owners from private land that in some cases had provided wildlife conservation. FTLRP aimed at revising private land tenure for white owners [24] but resulted in some degree of lawlessness. It was commonly referred to as *jambanja* (or violence) or land invasion [25], terms that indicate how the land was seized. At the time of the study, approximately 6,000 people lived in the new settlement established under the FTLRP in the southern portion of the SVC, which was historically a private wildlife refuge [26]. Because the resettlement was politically motivated and poorly planned, it resulted in the haphazard resettlement of large areas of private land [26]. Despite substantial research of this FTLRP on the people's socioeconomic status [24, 27, 28], its impact on HWCs and wildlife species conservation remains poorly understood.

The attitudes of people toward problem animals and in particular HWCs when land rights shift from private to communal ownership or when land ownership and resource tenure are unclear are poorly understood. Thus, this study will be important in evaluating residents' attitudes in a changing political and policy context through the unique lens of extreme measures of land redistribution, i.e., FTLRP. The objectives of this study were (i) to determine the key wildlife species causing conflict in SVC, (ii) to assess the perceptions of residents toward the major causes of conflict with wildlife, and (iii) to evaluate the attitudes of residents toward problem animals. We tested four hypotheses: (1) that illegal human resettlement into SVC is a major cause of HWCs as it increases encounters between wildlife and humans, (2) increased encounters will be correlated with more negative attitudes toward problem animals, (3) perceptions of increased encounters and negative attitudes toward conservation will be associated with increased preferences to capture and kill problem wildlife, and (4) that perception will differ according to the sociodemographic status of the resettled farmers.

## 2. Materials and Methods

### 2.1. Study Area

The study was conducted in human communities living inside the SVC which is located in the Southeast Lowveld of Zimbabwe. The SVC is located in agroecological region V of Zimbabwe characterized by high mean daily maximum temperatures of 35°C with rainfall ranging from 300 to 500 mm per annum and poor soil quality [29]. Surface water is limited with many river flows being subsurface during the dry season. All these conditions limit agricultural activity making wildlife ranching a more sustainable option.

SVC was established as a private game reserve and as a cooperatively managed wildlife area in 1991 from former cattle ranches [29]. Its establishment was motivated by the need to conserve a large stock of black rhinos (*Diceros bicornis*) that were translocated to the area by the government of Zimbabwe as part of its conservation scheme [30]. After the conversion of SVC from cattle to wildlife ranching, trophy hunting became the major economic activity [29]. SVC is home to the big five; buffalo (*Syncerus caffer*), elephant, lion, leopard, and black rhino; medium body-sized herbivores such as the greater kudu (*Tragelaphus strepsiceros*), and small-sized herbivores such as impala (*Aepyceros melampus*) and common duikers (*Sylvicapra grimmia*) while carnivores are dominated by the lion, spotted hyena, and African wild dog (*Lycaon pictus*) [26]. The major problem animals in the area are elephants, lions, and spotted hyenas [5]. Other animals that cause problems are chacma baboons (*Papio ursinus*), buffalo, and Nile crocodiles.

SVC is the largest private wildlife reserve in Africa [29] and originally covered an area of 3,490 km^2^ but was reduced to 2,530 km^2^ after an area measuring approximately 960 km^2^ was reallocated to subsistence communal farmers as part of the government of Zimbabwe's FTLRP in 2000 and 2001 [26]. Although the government of Zimbabwe decided to keep the SVC for wildlife management, some of its parts are still being used for subsistence farming. This has created a mosaic of wildlife and human habitat creating conditions for HWCs.

Before 1990, the area now called SVC was a cattle ranch although its establishment as a private wildlife conservancy was politically controversial because it had no statutory definition in Zimbabwean law [26]. It was formed through the signing of a constitution with the need for cooperative wildlife management while ensuring the sovereignty of the ranches. In addition, the government of Zimbabwe has always held the conservancy in suspicion due to the dominance of the sector by white commercial farmers who are viewed as former colonialists [31]. The conservancy was regarded by many politicians as an attempt by these farmers to privatize wildlife. This was understandable since the conservancy model which gave land rights to the owners challenged the government's control of wildlife in state-protected areas. Land tenure, therefore, is an important factor for wildlife species conservation because it entails property rights [32]. Thus, the ranches were owned by individuals although the wildlife resources were managed cooperatively. These rights shifted with the advent of the FTLRP as private land was reallocated to become communal land through resettlement.

At the inception of FTLRP in 2000, the game fence around SVC was stolen facilitating the access of poachers and providing material to make snares [29]. For example, poaching rates increased dramatically after the FTLRP; between August 2001 and June 2009 over 84,000 snares were removed and 4,148 poachers were arrested [33]. Carcasses or remains of poached animals recovered during the same period amounted to 6,454 [33]. This demonstrates the lawlessness that is associated with political instability. The removal of the fence increased encounters between residents and wildlife as the latter were now free-roaming.

### 2.2. Research Design and Sampling

**Figure 1:**
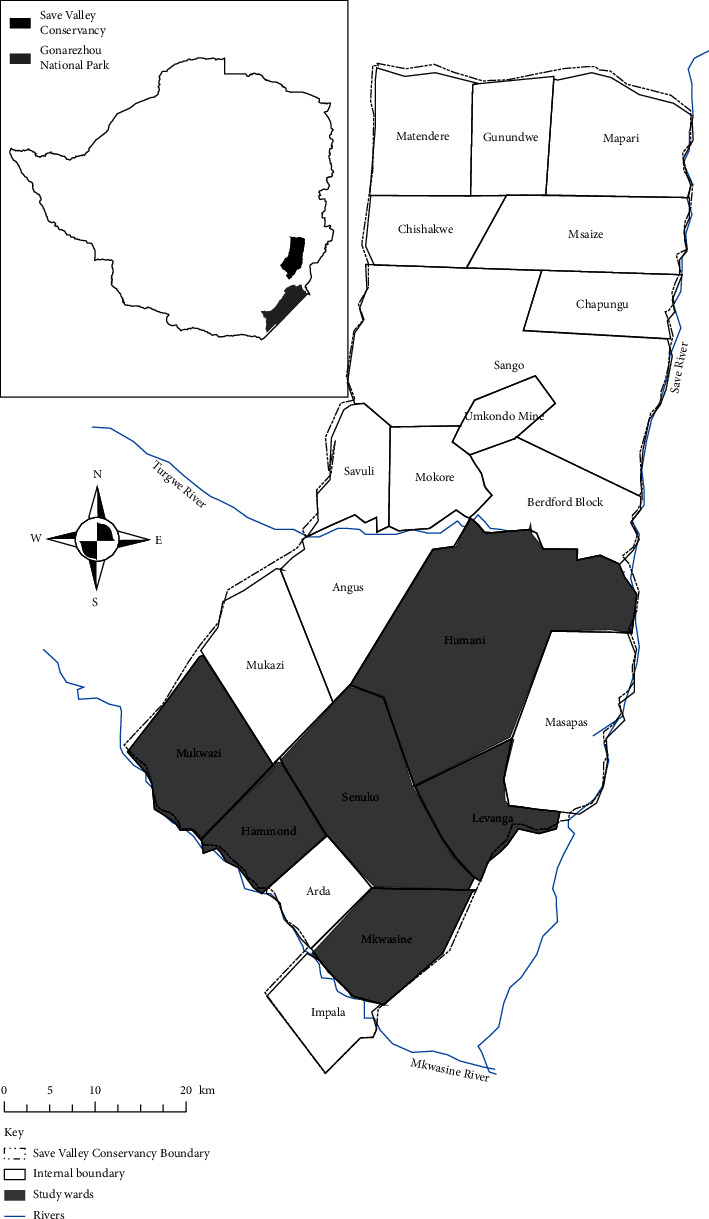
Map of the study area.

SVC was originally made up of 24 individual properties, although this constitutes 18 management units or extensive ranches. The study was conducted in six of these ranches found in Ward 24 namely Levanga, Humani, Hammond, Mkwasine, Mkwazi, and Senuko (shaded on the map, Figure 1). After a preliminary survey, Ward 24 was purposively selected because it is both a resettled area and has reported cases of HWCs. The southern part of SVC is currently being used for both wildlife production and subsistence farming, a precondition for HWCs [29]. The population for the study area is comprised of 600 households which include 50 professionals and 550 SVC community members. Within the ward, 290 respondents were randomly selected from six farms that make up the ward. In the community register, each household had a corresponding number according to the listing from 1 to 600. Random numbers generated in Microsoft Excel were then assigned to each household. Households with a corresponding random number were then picked and sampled. This was done to afford an equal chance of picking households. Two local villagers in each ranch assisted in locating each household. At each household, the household head or the oldest family member present was interviewed.

### 2.3. Data Collection

Face-to-face semistructured interviews and two field reconnaissance visits were conducted among the six ranches in ward 24 from January 2014 to June 2014. Respondents were asked to list the root causes of HWCs. They were then asked to rate the causes as major causes on a scale of 1 (strongly disagree) to 5 (strongly agree).

Attitudes toward problem-causing animals were assessed by asking four scenario questions: (1) What action would you take if you encounter wildlife? (2) What action would you take if the problem animal kills livestock (depredation)? (3) What action would you take if the problem animal raids crops? (4) What action would you take if the problem animal kills a person? The responses were then partitioned into three categories: positive attitude, negative attitude, and neutral attitude as described below. The response was classified as positive if (a) the person reports to authority, (b) the person scares the animal using nonlethal methods such as bells or beating tins or any other nonlethal method, and (c) the person traps the animal and reports to authority, e.g., cage trap a snake or small carnivore. The response was classified as negative if the person kills the animal or if the person traps or snares the animal intending to kill it. The response was neutral if the person takes no action or if the person runs away with no subsequent action. However, if the person runs away and their subsequent action falls under positive or negative action, it was recorded under those attitudes accordingly.

The socioeconomic status of each respondent was determined by recording their sex (male or female), education (no formal education, primary, secondary, or college), age (<30 years, 31–40 years, or >40 years), and period of stay in the area (<5 years or >5 years). Respondents were asked to identify the major problems animals and the associated problems they cause. The interview questions were pilot tested in a nearby ward, the data from which was not included in the present study.

In March and April 2014, two field surveys were conducted to verify and correctly interpret the results of the semistructured interviews. The field surveys involved visiting fields and kraals where problem animals had been reported. Assessments included taking photographs as a way of interpreting the level of damage that would have been caused. The complementary field surveys were conducted to triangulate information as respondents are known for giving incorrect responses.

### 2.4. Data Analysis

The causes of HWCs were grouped into seven categories: (1) change in land use, (2) land fragmentation, (3) competition for natural (water and pasture) resources, (4) lack of capacity, (5) contested land ownership, (6) dislike of Pas, and (7) overpopulation, by identifying themes from the collected data and employing a coding system. First, we assigned a numeric code that identified the cause of conflict. We then hand-coded each interview assigning each response to emerging and recurrent themes as part of an indexed text-based dataset [34]. In this way, we arrived at seven causes that emerged from the responses of the residents. Each of these causes was ranked as the major cause on a scale of 1 (strongly disagree) to 5 (strongly agree) according to the respondents' opinion.

We analyzed the causes of HWCs using an ordinal logistic regression (OLR) model. The OLR model was used because the responses followed an ordinal scale (1 = strongly disagree to 5 = strongly agree) which we now call the ‘categories' in the analyses. Since the categories were evenly distributed, we used a logit link function. When performing the analysis, the categories were taken to be the threshold components while the sociodemographic factors: sex (male or female), education (no formal education, primary, secondary, or college), age (<30 years, 31–40 years or >40 years), and period of stay in the area (<5 years or >5 years) were taken to be the location component.

We used a multinomial logistic regression (MLR) model to predict the attitude of respondents toward problem animals in each sociodemographic category: sex, education, age, and period of stay in the SVC. The attitude of the respondent (negative, positive, or neutral) was taken to be the dependent variable while the sociodemographic factors were taken to be the independent variables.

In all the models, we included all the socioeconomic variables and used backward stepwise elimination of nonsignificant variables (*p* > 0.05 eliminated) and remained with those variables that represented the minimal adequate model. Likelihood ratio tests (LRTs) were used to test the significance of each predictor. Parameter estimates (*B*), Wald statistics, and odds ratios (OR) were used to compare the relative effects of the reference category to the dependent variables of interest. The predictor variables were tested for multicollinearity and the test showed a Variance Inflation Factor (VIF) of <1.7 indicating that the intercorrelation levels were appropriate for analysis. All analyses were conducted in the statistical package IBM Statistical Package for the Social Sciences (SPSS) version 21.

## 3. Results

### 3.1. Demographic Characteristics of Respondents

Participants in this study were mainly subsistence farmers who had stayed in the area for less than five years. Most of them were young respondents (<30 years) who had attained at least primary education. There was approximately gender balance among the respondents although females were slightly more than male respondents (Table 1).

### 3.2. Problem-Causing Animals

Most of the respondents were of the view that the major problem animals in SVC causing depredation were lions (98% of respondents), hyenas (93%), leopards (53%), and crocodiles (60%) with elephants being the major crop raiders (100%) and involved in the human attack (98%). Crocodiles (cited by 98% of respondents) and lions (cited by 80% of respondents) were also identified as major culprits in human attacks.

### 3.3. Perceptions of Respondents on the Causes of HWCs

Our OLR model predicting the major causes of HWCs was significant for age group (Wald *χ*^2^ = 24.01; d*f* = 4; *p*=0.002), education (Wald *χ*^2^ = 54.01; d*f* = 6; *p* ≤ 0.0001) and period of stay in the SVC (Wald *χ*^2^ = 65.14; d*f* = 2; *p* ≤ 0.0001) and sex (Wald *χ*^2^ = 28.38; d*f* = 2; *p*=0.001). All the interactions were not significant (*p* > 0.05).

Respondents who had lived in the area for more than 5 years were more likely to agree that change in land use (*B* = 1.32, OR = 3.74) and contested land ownership (*B* = .67, OR = 1.95) are the major causes of HWCs than those who had lived in SVC for less than 5 years. Respondents of younger age groups were more likely to strongly agree that changes in land use (*B* = 1.57, OR = 4.81 for <30 years and *B* = 1.82, OR = 6.17 for 31–40 years) were the major causes of HWCs than those of greater than 40 years. Having primary (*B* = −1.85), secondary (*B* = −1.87), or college (*B* = −1.75) decreased the likelihood of agreeing that change in land use is the major cause of HWCs compared to respondents with no formal education. On the other hand, respondents with formal education were more likely to strongly agree that contested land ownership is the major cause of HWCs (Table 2).

### 3.4. Attitudes of Respondents toward Problem Animals

The MLR model predicting the attitude of respondents toward wildlife species conservation was significant for sex (*χ*^2^ = 21.49; d*f* = 3; *p*=0.002; Nagelkerke pseudo *R*^2^ = 0.74), education (*χ*^2^ = 11.79; d*f* = 3; *p*=0.008; Nagelkerke pseudo *R*^2^ = 0.75) and period of stay in the SVC (*χ*^2^ = 9.06; d*f* = 1; *p*=0.003; Nagelkerke pseudo *R*^2^ = 0.73), but not for age (*χ*^2^ = 2.06; d*f* = 2; *p*=0.08). Males were less likely to have a negative attitude toward problem animals compared to females (*B* = −1.39; OR = .25). Having no formal education increased the likelihood of having a negative attitude toward problem animals compared to those with primary education (*B* = 1.75, OR = 5.75), secondary education (*B* = .94, OR = 2.56), and college education (*B* = 1.39; OR = 4.01). There was a positive association between the period of stay in the SVC and the attitude of respondents with those that have stayed for less than 5 years more likely to have a negative attitude toward problem animals than those who have stayed for long (*B* = 3.6; OR = 36.71, Table 3).

## 4. Discussion

### 4.1. Problem-Causing Wildlife Species

Our results showed that key wild animals causing conflicts in SVC were elephants, lions, hyenas, and to a lesser extent leopards and crocodiles. Gandiwa et al. [16] also showed that lions, elephants, and hyenas were among the most damage-inflicting animals in communities living adjacent to northern Gonarezhou National Park, Zimbabwe. Our study is also consistent with that of Matseketsa et al. [5] who showed that lions, hyenas, and elephants were the most troublesome animals in SVC. Large-bodied herbivores and carnivores tend to have large home ranges due to their high energy demand and therefore need abundant food to survive [35]. So in addition to foraging on wild species, carnivores also target livestock while herbivores feed on crops leading to increases in HWCs.

Furthermore, the successful conservation efforts of elephants and lions have led to their population increase. This wildlife population increase plus the ever-increasing human population has led to accelerated competition for resources between wild animals and humans [36–38]. Predators occur in areas with high human population growth, potentially accelerating conflict rates [39]. In addition, in the current study, resettled farmers were located in wildlife areas which may have increased HWCs. These findings are consistent with other studies in Zimbabwe on HWCs, which found that carnivores are the biggest problem animals around PAs [5, 26, 37, 40].

### 4.2. Respondents' Perceptions of the Causes of HWCs

In general, residents perceived that land-use change at SVC was the primary cause of HWC, confirming our hypothesis that human relocation to wildlife areas is a major cause of HWC because it leads to more human-wildlife encounters. Perceptions also varied with the sociodemographic status of respondents. Residents who have lived in SVC longer felt that changing land use and disputed land ownership were the main causes of HWCs. Indeed, land use at SVC has changed from predominantly wildlife ranching to mixed agriculture (wildlife ranching and subsistence farming). When the government implemented the FTLRP, people were settled within a wildlife area, which increased their contact with wildlife. In addition, the fence between communities and the SVC was knocked down by settlers, allowing wildlife to roam freely, which created favorable conditions for HWCs.

Lack of proper land use planning and increasing competition for space and resources have been cited as major causes of HWCs [41]. In the current study, competition for resources was considered one of the main causes of HWCs by respondents with at least primary education. Studies have also shown that the settlement of people inside or near PAs increases HWCs [40, 42]. The relocation of people to SVC was politically motivated and poorly planned, without a clear strategy on how to ensure the sustainable protection of wildlife species. The main goal of land reform was productivity (crop yields to avoid hunger) and to address political imbalances created by the previous white regime. However, there was little to no evidence that environmental impact assessments were conducted before the implementation of the FTLRP. This resulted in poorly planned resettlement, some of which occurred on wildlife corridors and former wildlife habitats. In addition, the four resettlement programs adopted in Zimbabwe (*A*1, *A*2, *A*3 Animal, and the cooperative) did not fully promote wildlife-human coexistence. This was exacerbated by the fact that most of the resettled farmers continued to practice subsistence agriculture, which subsequently created a conducive environment for crop depredation and increased HWCs [25].

Period of residence in the SVC led to differing views on the protection of wildlife. Residents who had lived in the region for fewer years had more negative perceptions of crop-raiding and wildlife depredation than those who had settled earlier. However, encounters with wildlife did not elicit a negative reaction toward wildlife. Research has shown that negative attitudes toward protecting certain wildlife species do not always equate to behaviors such as retaliatory killing [43]. It becomes problematic when the animal interferes with the needs of residents, such as through depredation and crop-raiding. This aligns well with Mutanga et al.'s [44] study in northern Gonarezhou National Park in Zimbabwe, which found that people who had lived near the study area for fewer years had negative attitudes toward problem animals. When someone encounters an animal and the animal does not harm the person, it does not usually lead to HWCs. However, the negative attitudes were only triggered when residents felt unsafe or when wildlife affected their quality of life and food security status.

Many residents relocated to SVC during the FTLRP in 2000 began to engage in rampant poaching of wildlife and firewood [21], which may have shaped their attitudes toward problem animals. According to Le Bel et al. [40], immigrants to conservation areas are less likely to tolerate wildlife species that they view as competitors. However, King [45] found that new immigrants are more likely to support wildlife conservation when they are less dependent on natural resources for their livelihood. In this study, the immigrants at SVC were poor small-scale farmers who made their living from cultivating small areas of cereal crops. Thus, if they lose their livelihoods to wildlife crop-raiding, they are likely to develop negative attitudes. Most of them had less than five heads of livestock. So, if only one animal is lost, it means a significant loss of livelihood and justifies their strong negative attitude toward problem animals. In contrast, residents who had lived in and around SVC for a longer period did not view depredating animals as negatively, probably because they had developed measures to reduce the impact of depredation whereas the new immigrants were still adjusting to the new situation.

### 4.3. Respondents' Attitudes toward Problem Animals

Results showed that males were less likely to have negative attitudes toward problem animals compared to females, while a lack of formal education increased the likelihood of negative attitudes toward problem animals compared to those with primary education. According to Arjunan et al. [46], attitudes toward wildlife may also be influenced by gender. In this study, gender did not influence perceived causes of HWCs. However, attitudes toward problem animals were correlated with gender, with males having more positive attitudes than females. The influence of gender on risk perception is dynamic and complex, and studies of gender perception in wildlife conservation are inconclusive. It has been shown that men and women may perceive and interpret an apparently identical risk differently [47]. As a result, gender-specific questions tend to be context-specific, so results are likely to vary across study areas and aspects examined. In our study context, the lack of gender differences in perceived causes of HWCs can be attributed to similar patterns of community livelihoods. Both men and women reported going to the forest, albeit for different reasons. Men go to herd cattle, while women go to fetch firewood. This suggests that the cost of HWCs was borne equally by both sexes. Consistent with our study, Kideghesho et al. [48] showed that gender did not influence residents' perceptions of wildlife species conservation.

In support of our findings, Alexander et al. [49] and Mutanga et al. [44] showed that gender influences beliefs and attitudes, with women having more negative attitudes toward wildlife. It has been suggested that because women are more dependent on the ecosystem for their livelihood, they are likely to bear more costs associated with HWCs [50]. In addition, women tend to fear predators more than men because they bear more responsibility for household security [49, 51]. This fear may be exacerbated by reports of human deaths near PAs. For example, the Zimbabwe Parks and Wildlife Authority, the government agency responsible for managing wildlife, reported that nine people died from wildlife attacks between January and December 2014 at SVC [52]. Such statistics trigger fear among residents, who then develop negative attitudes toward wildlife. Other residents said they were willing to band together to kill the animals involved in attacks on humans. However, one of the animals involved in attacks on humans, the elephant, cannot be killed easily with the inferior weapons of the residents. This inability to control elephants reinforces the fear of wild animals and negative attitudes. In some cases, residents overcome this challenge by poisoning the elephants with cyanide, a trend that has increased in Zimbabwe's PAs.

Our results showed that residents with at least a university education had positive attitudes when encountering problem animals. High levels of understanding and reasoning among the educated are important factors in supporting wildlife conservation [51]. This is consistent with our study, which found that the likelihood of tolerating wild species increased with higher levels of education. Most of the positive attitude in this study was because many educated respondents would call the authorities if a problem animal raided crops or killed livestock, while those without formal education were mainly involved in retaliatory killings. Conservation education can reduce hostility toward wildlife, and educated communities are more receptive to such education programs. In addition, educated community members are likely to find better jobs and alternative livelihoods in nearby areas, reducing their dependence on natural resources [53]. Elsewhere, Alexander et al. [49] found that education did not influence community attitudes toward PAs, although the study focused on pastoralists rather than entire communities.

Sometimes education does not influence people's attitudes when they are shaped by traditional beliefs rather than formal education. The relocated residents in the current study had diverse educational backgrounds. For example, most of the residents relocated to SVC were not from surrounding areas and likely received their education in their community of origin. In some of these communities, conservation and wildlife management issues are not given much attention because the communities are located far from wildlife refuges. As a result, these people are unlikely to tolerate wildlife damage and loss.

## 5. Conclusion

To understand the main causes of HWCs, one needs to know the attitudes of people toward problem animals in a particular context. This study was conducted in a context where farmers had been resettled in wildlife areas under a violent Fast Track Land Reform Program. Our study found that the change in land use due to resettlement was seen as the main cause of HWCs in the region in SVC. The change in land use created a mosaic of wildlife and human habitat, which is a possible driver for HWCs. This result confirms our initial hypothesis that the relocation of people to SVC is a major cause of HWCs and that perceptions of increased encounters correlate with more negative attitudes toward problem animals. Residents also felt that depredation by lions, hyenas, and leopards and crop-raiding by elephants were the main causes of HWCs. The results showed that residents were more likely to kill wildlife if they had either killed a human or were involved in depredations. This is consistent with our hypothesis that perceptions of increased encounters and negative attitudes toward problem animals are associated with an increased preference for capturing and killing problem wildlife species.

Our results show that residents' attitudes toward wildlife depend on sociodemographic factors. Residents who had been settled for fewer years had more negative attitudes toward crop-raiding and depredation of wildlife than those who had settled earlier. This could be due to their low appreciation of the value of wildlife because they cannot coexist with wildlife as the time frame did not allow for meaningful integration. Level of education correlated with attitude toward problem animals, with the more educated having a positive attitude. The results confirm our hypothesis that perceptions differ according to the sociodemographic status of resettled farmers.

## 6. Recommendations

This study has implications for conservation and policymaking in wildlife-protected areas. Since the resettlement of people into SVC did not provide a framework of coexistence between people and wildlife, the government has to look at its FTLRP schemes and incorporate issues of HWCs into their plans. Since the land-use change was found to be the major driver of HWCs, we further recommend that there is a need to regularize the settlement of farmers in SVC so that residents are not haphazardly settled. This may go a long way in fighting HWCs in the area as people will be living in planned settlements. Coordinating HWCs and implementing sustainable conservation objectives are easy in a well-planned settlement.

Given that residents' perceptions of HWCs varied with sociodemographic factors, we recommend that efforts to mitigate HWCs should incorporate people of different social statuses. Most importantly, residents who were resettled later into the SVC need to be educated on HWCs issues so that they develop a positive attitude toward problem animals. In addition, some of these recent migrants should be incorporated into community structures involved in wildlife conservation so that they learn the concepts of wildlife conservation in the context of HWCs. Finally, further studies should be carried out to compare results across different areas that have undergone FTLRP to obtain a better understanding of human perceptions and HWCs across Zimbabwe.

## Figures and Tables

**Table 1:** Demographic characteristics of respondents from the Save Valley Conservancy survey from January to June 2014.

Variable	Number of participants (% in parentheses)
*Sex*	
Male	131 (45%)
Female	159 (55%)

*Age group*	
<30 years	145 (50%)
31–40 years	102 (35%)
>40 years	43 (15%)

*Education*	
No formal education	44 (15%)
Primary	93 (32%)
Secondary	102 (35%)
College	51 (18%)

*Occupation*	
Farmer	241 (83%)
Other	49 (17%)

*Period of stay*	
<5 years	209 (72%)
>5 years	81 (28%)

**Table 2:** Parameter estimates of variables in the ordinal logistic regression (OLR) model used to predict the residents' perceptions of the major causes of HWCs in Save Valley Conservancy. The parameter “dislike of protected areas” (*n* = 34) was taken to be the reference category for comparisons.

Explanatory variables	Change of land use (*n* = 70)		Land fragmentation (*n* = 52)		Competition for resources (*n* = 26)		Lack of capacity (*n* = 20)		Contested land ownership (*n* = 65)		Overpopulation (*n* = 23)	
	*B*	OR	*B*	OR	*B*	OR	*B*	OR	*B*	OR	*B*	OR
Sex: female vs male	−1.42	0.24^*∗∗*^	0.98	2.66	0.34	1.40	0.23	0.79	−1.72	0.18^*∗∗*^	1.34	3.82
Period of stay in SVC: <5 years vs >5 years	1.32	3.74^*∗∗∗*^	−0.45	0.64	−0.23	0.79	−0.52	0.59^*∗∗*^	0.67	1.95^*∗∗∗*^	0.45	1.57
Age: <30 years vs >40 years	1.57	4.81^*∗∗*^	−0.45	0.64	0.67	1.95	−1.85	0.16^*∗∗*^	−1.41	0.24^*∗∗*^	0.25	1.28
Age: 31–40 years vs >40 years	1.82	6.17^*∗∗*^	0.67	1.95	−0.23	0.79	−1.99	0.14^*∗∗*^	−1.44	0.24^*∗*^	0.13	1.14
Education: primary vs none	−1.85	0.15^*∗∗*^	−1.09	0.34^*∗∗∗*^	−1.68	0.19	−0.75	0.47^*∗∗*^	1.42	4.14^*∗∗*^	−1.09	0.34^*∗∗∗*^
Education: secondary vs none	−1.87	0.15^*∗∗∗*^	−1.68	0.19^*∗∗∗*^	−1.15	0.32	−1.84	0.16^*∗∗∗*^	1.66	5.26^*∗∗*^	−1.30	0.27^*∗∗∗*^
Education: college vs none	−1.75	0.17^*∗∗∗*^	−1.61	0.20^*∗∗∗*^	−1.56	0.21	−0.91	0.40^*∗∗∗*^	0.89	2.44	−0.48	0.62^*∗∗∗*^

^*∗*^, ^*∗∗*^, and ^*∗∗∗*^ indicate the significance of parameters at 10%, 5%, and 1%, respectively; *B* = regression coefficient and OR = odds ratio. For age group, “>40 years” and for education, “none” were taken to be the reference categories for comparison.

**Table 3:** Parameter estimates of variables in the multinominal logistic regression (MLR) model used to predict local people's attitudes toward wildlife species conservation in Save Valley Conservancy. Parameters for “negative attitude” (*n* = 114) and “positive attitude” (*n* = 78) are presented while parameters for the “neutral attitude” (*n* = 98) were taken to be the reference category for each of the scenarios.

Explanatory variables	Category	Positive attitude			Negative attitude		
		*B*	SE	OR	*B*	SE	OR
Sex	Female vs male	−0.09	0.43	0.91^*∗*^	−1.39	0.79	0.25^*∗∗*^
Age group	<30 years vs >40 years	1.59	1.10	4.90	1.48	1.07	4.40
	31–40 years vs >40 years	0.90	1.21	2.46	0.47	1.23	1.60
Education	Primary vs none	−0.65	0.57	0.52	1.75	1.31	5.75^*∗∗∗*^
	Secondary vs none	−0.46	0.55	0.63	0.94	0.90	2.56^*∗∗∗*^
	College vs none	−2.86	1.13	0.06^*∗∗*^	−1.39	0.12	4.01^*∗∗∗*^
Period of stay in SVC	<5 years vs >5 years	0.02	1.21	1.02	3.60	1.43	36.71^*∗∗*^

^*∗*^, ^*∗∗*^, and ^*∗∗∗*^ indicate the significance of parameters at 10%, 5%, and 1%, respectively; *B* = regression coefficient and OR = odds ratio; SE = standard error. For age group, “>40 years” and for education, “none” were taken to be the reference categories for comparison.

## Data Availability

The data used to support the findings of this study are available from the corresponding author upon request.
